# Validation of methods to identify people with idiopathic inflammatory myopathies using hospital episode statistics

**DOI:** 10.1093/rap/rkac102

**Published:** 2022-12-02

**Authors:** Jennifer R Hannah, Patrick A Gordon, James Galloway, Megan Rutter, Emily J Peach, Michael Rooney, Peter Stilwell, Matthew J Grainge, Peter C Lanyon, Mary Bythell, Fiona A Pearce

**Affiliations:** Centre for Rheumatic Diseases, King's College London, London, UK; Department of Rheumatology, King's College Hospital, London, UK; National Congenital Anomaly and Rare Disease Registration Service, NHS Digital, London, UK; Centre for Rheumatic Diseases, King's College London, London, UK; Department of Rheumatology, King's College Hospital, London, UK; Centre for Rheumatic Diseases, King's College London, London, UK; Department of Rheumatology, King's College Hospital, London, UK; National Congenital Anomaly and Rare Disease Registration Service, NHS Digital, London, UK; Lifespan and Population Health, University of Nottingham, Nottingham, UK; Department of Rheumatology, Nottingham University Hospitals NHS Trust, Nottingham, UK; Lifespan and Population Health, University of Nottingham, Nottingham, UK; Department of Rheumatology, King's College Hospital, London, UK; National Congenital Anomaly and Rare Disease Registration Service, NHS Digital, London, UK; Lifespan and Population Health, University of Nottingham, Nottingham, UK; National Congenital Anomaly and Rare Disease Registration Service, NHS Digital, London, UK; Lifespan and Population Health, University of Nottingham, Nottingham, UK; Department of Rheumatology, Nottingham University Hospitals NHS Trust, Nottingham, UK; National Congenital Anomaly and Rare Disease Registration Service, NHS Digital, London, UK; National Congenital Anomaly and Rare Disease Registration Service, NHS Digital, London, UK; Lifespan and Population Health, University of Nottingham, Nottingham, UK; Department of Rheumatology, Nottingham University Hospitals NHS Trust, Nottingham, UK

**Keywords:** myositis, rare diseases, epidemiology, ICD-10, hospital episode statistics, findability

## Abstract

**Objective:**

Hospital episode statistics (HES) are routinely recorded at every hospital admission within the National Health Service (NHS) in England. This study validates diagnostic ICD-10 codes within HES as a method of identifying cases of idiopathic inflammatory myopathies (IIMs).

**Methods:**

All inpatient admissions at one NHS Trust between 2010 and 2020 with relevant diagnostic ICD-10 codes were extracted from HES. Hospital databases were used to identify all outpatients with IIM, and electronic care records were reviewed to confirm coding accuracy. Total hospital admissions were calculated from NHS Digital reports. The sensitivity and specificity of each code and code combinations were calculated to develop an optimal algorithm. The optimal algorithm was tested in a sample of admissions at another NHS Trust.

**Results:**

Of the 672 individuals identified by HES, 510 were confirmed to have IIM. Overall, the positive predictive value (PPV) was 76% and sensitivity 89%. Combination algorithms achieved PPVs between 89 and 94%. HES can also predict the presence of IIM-associated interstitial lung disease (ILD) with a PPV of 79% and sensitivity of 71%. The optimal algorithm excluded children (except JDM code M33.0), combined M33.0, M33.1, M33.9, M36.0, G72.4, M60.8 and M33.2, and included M60.9 only if it occurred alongside an ILD code (J84.1, J84.9 or J99.1). This produced a PPV of 88.9% and sensitivity of 84.2%. Retesting this algorithm at another NHS Trust confirmed a high PPV (94.4%).

**Conclusion:**

IIM ICD-10 code combinations in HES have high PPVs and sensitivities. Algorithms tested in this study could be applied across all NHS Trusts to enable robust and cost-effective whole-population research into the epidemiology of IIM.

Key messages

- Administrative National Health Service databases are powerful, pre-existing and under-utilized epidemiological resources for rare diseases.
- Our recommended optimal algorithm for identification of idiopathic inflammatory myopathies has 89% positive predictive value and 84% sensitivity.
- Idiopathic inflammatory myopathy-associated interstitial lung disease is identified by respiratory ICD-10 codes with 79% positive predictive value and 71% sensitivity.

## Introduction

Treatment of patients with rare diseases is challenging because of their low prevalence and the scarcity of knowledge and expertise [1]. Yet, there are >6000 known rare diseases, collectively affecting between 6 and 8% of the population [2]. Understanding more about these diseases is a public health priority [3]. The National Congenital Anomaly and Rare Disease Registration Service (NCARDRS) aims to achieve population-based registration of all patients in England with congenital or rare diseases in order to support improvements in understanding and outcomes for these conditions. Within NCARDRS, the Registration of Complex Rare Diseases—Exemplars in Rheumatology (RECORDER) project is specifically seeking new methodologies for registering rare rheumatological diseases, such as the idiopathic inflammatory myopathies (IIMs), using routinely collected health-care data.

IIM describes a group of chronic, autoimmune rheumatological conditions, most commonly sub-classified as PM, DM, JDM and IBM [4]. They manifest with varying levels of systemic and localized inflammation, primarily of the skeletal muscles, skin and lungs [5]. Interstitial lung disease (ILD) is a common complication that affects ∼40% of patients [6]. Previous estimates have placed the prevalence of IIMs between 2.4 and 33.8 individuals per 100 000 and the incidence between 1.16 and 19 new cases per million patient-years [7].

Hospital episode statistics (HES) are routinely recorded data from every hospital admission within the National Health Service (NHS) in England, including private patients treated in NHS hospitals and activity of NHS-funded treatment centres [8]. For every continuous episode of inpatient or day-case care under a single consultant and hospital provider, a record is generated containing demographic and diagnostic information, termed a finished consultant episode. For simplicity, finished consultant episodes are henceforth referred to as admissions.

The primary reason for admission and any co-morbidities are recorded using ICD-10 codes [9]. HES data provide a unique research opportunity to describe national admission patterns and infer information regarding the incidence and prevalence of disease in conditions that require secondary care input.

The usefulness of HES data for epidemiological research depends on coding accuracy. Coders are nationally trained and follow national coding procedures in order to reduce variation [10, 11]. Within other rare rheumatological diseases, studies of coding accuracy in HES have found positive predictive values (PPVs) for Kawasaki disease, ANCA-associated vasculitis and Takayasu’s arteritis of 100, 92 and 91.7%, respectively [12]. However, codes for adult-onset Still’s disease, relapsing polychondritis and polyarteritis nodosa performed poorly, with respective PPVs of only 42.8, 40.0 and 5.0% [12]. Accuracy of coding is therefore highly disease dependent. A Norwegian study used ICD-10 codes in IIM to assess the incidence and prevalence of IIM in Norway, and a UK study used ICD-10 codes alongside other identification methods to calculate the incidence; however, there has not previously been any validation for this technique within HES data [13, 14].

The aim of this study was to validate the use of ICD-10 codes within HES as a method of accurately identifying patients with an IIM by comparison with local clinical records.

## Methods

This retrospective study is reported using Standards for Reporting of Diagnostic Accuracy Studies (STARD) guidelines for reporting diagnostic accuracy studies [15]. ICD-10 codes related to neuromuscular disorders were identified and consensus agreement for inclusion/exclusion reached by five co-authors with specialist clinical experience of IIM patients (Supplementary Table S1, available at *Rheumatology Advances in Practice* online).

All patients allocated any pre-identified diagnostic codes for IIM in primary or secondary diagnoses fields of HES from 6 April 2010 to 5 April 2020 for admitted patient care at King’s College Hospitals NHS Trust (KCH) were identified. KCH covers five hospital sites across a large urban area and runs a regional tertiary myositis service. A locally kept rheumatology database used for all outpatient documentation was searched simultaneously for all outpatients with IIM during the same time period. Additionally, records of all patients undergoing myositis-specific autoantibody testing (a routine part of IIM patient assessment) were searched manually for additional IIM cases to pick up occasional patients known only to other specialties or who had died before outpatient attendance. The study population was created by combining the HES and local datasets.

Given the high rates of ILD complicating IIM, co-occurring ILD codes were also extracted. Co-existence of additional exclusion codes that might make a diagnosis of IIM less likely were recorded.

The total number of HES admissions at the NHS Trust over the same time period was calculated using publicly available data from NHS Digital.

Case notes were reviewed by expert IIM clinicians to ensure that all patients identified had a clinically probable diagnosis of IIM or IIM-associated ILD. Clinicians were blinded to the results of the HES extract. The sensitivity, specificity, negative and positive predictive values (NPV and PPV) of each individual ICD-10 diagnostic code for IIM were calculated, with the total population taken as the total number of admissions at the Trust over the time period.

**Figure 1.**
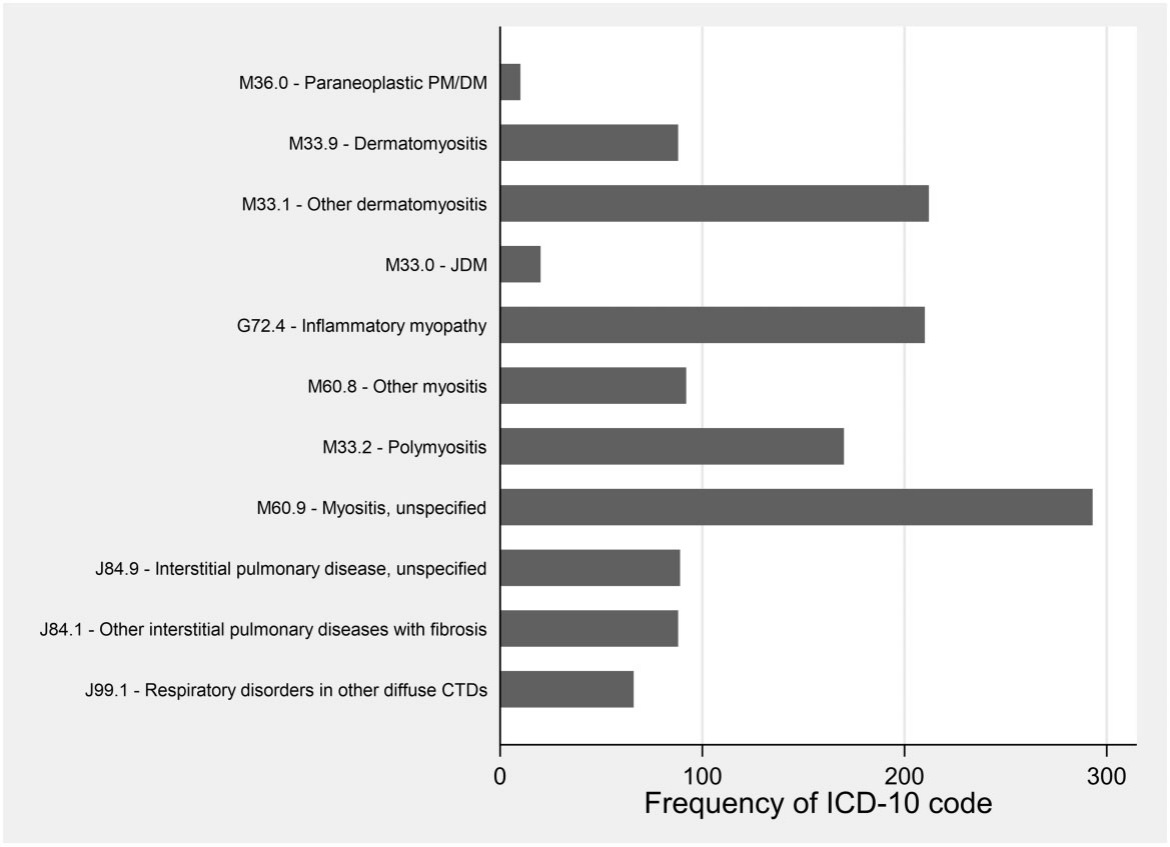
Frequency of idiopathic inflammatory myopathy-related hospital episode statistics codes from King’s College Hospitals NHS Trust-submitted coding data 2010–2019, listed in decreasing order of positive predictive value. Note that interstitial lung disease codes were extracted only if co-occurring alongside a diagnostic code for idiopathic inflammatory myopathy

Various code algorithms (Fig. 1) were attempted to combine diagnostic codes for IIM and monitor the effect on sensitivity and PPV.

Where local notes were unavailable or insufficient to establish a clinical diagnosis, a negative assumption was made that IIM was not present. Additional sensitivity analysis was run, assuming any incomplete local data were positive for IIM, to assess the impact of this on results. Analysis of false negatives was performed to compare true positives with false negatives. Patient exposure years were calculated using a surrogate start date taken as the date of first appearance of the ICD-10 code, or for patients missing from HES, the date of the first outpatient visit. Linkage with the Mortality & Births Information System (MBIS) allowed calculation of the mortality incidence ratio. Age and sex were compared using a two-tailed *Z*-test or two-sample test of proportions, with the confidence level set at 95%.

Once optimal algorithms had been created, they were tested in a sample of 60 randomly selected cases in another NHS Trust.

Statistical analysis was undertaken using Stata v.17.0 and repeated in RStudio by a second independent researcher for quality assurance [16, 17].

Data for this study were collected and analysed under the National Disease Registries Directions 2021, made in accordance with sections 254(1) and 254(6) of the 2012 Health and Social Care Act, meaning that individual informed consent was not required. Further ethical approval for this study was not required per the definition of research according to the UK Policy Framework for Health and Social Care Research.

## Results

From 1 948 731 admissions at KCH between 2010 and 2020, 672 unique patients with prespecified diagnostic codes for IIM were identified. Fig. 1 demonstrates the relative frequency of each code. A further 61 patients not identified by HES with IIMs were found by interrogating local outpatient records.

After case note review, 510 of the 672 HES-identified patients were confirmed to have IIM, giving a PPV for all codes of 75.9% (95% CI 72.5, 79.1) and a sensitivity of 89.3% (95% CI 86.5, 91.7; i.e. 510 of 571 genuine IIM cases were identified). Twenty-one individuals were <16 years of age at the time of code allocation (excluding those with JDM code M33.0), with only one being a true IIM case. Excluding any code allocated while <16 years of age meant that 509 of 651 HES-identified patients were confirmed to have IIM, improving the PPV to 78.2% (95% CI 74.8, 81.3) without changing the sensitivity; therefore, children were excluded from all further analysis (except for JDM code M33.0). The effect of excluding children is shown in Table 1. Two-by-two tables are provided in Supplementary Fig. S1, available at *Rheumatology Advances in Practice* online.

**Table 1.** Positive predictive value and sensitivity of individual ICD-10 codes excluding patients <16 years of age (except for JDM codes) and the impact of including them

ICD-10 code	Description	Confirmed cases/code frequency (n/n)	PPV [% (95% CI)]	Sensitivity* [% (95% CI)]	Number of children excluded	Change in PPV by excluding children (%)	Change in sensitivity by excluding children (%)
M36.0	Paraneoplastic PM/DM	10/10	100.0 (69.2, 100.0)	1.8 (0.8, 3.2)	0	0.0	0.0
M33.9	DM	84/88	95.5 (88.8, 98.7)	14.7 (11.9, 17.9)	0	N/A	N/A
M33.1	Other DM	202/212	95.3 (91.5, 97.7)	35.4 (31.5, 39.5)	3	0.0	+0.5
M33.0	JDM	19/20	95.0 (75.1, 99.9)	3.3 (2.0, 5.2)	N/A	0.0	0.0
G72.4	Inflammatory myopathy	193/210	91.9 (87.4, 95.2)	33.9 (30.0, 37.9)	0	0.0	−0.1
M60.8	Other myositis	82/92	89.1 (80.9, 94.7)	14.4 (11.6, 17.5)	13	+11.0	0.0
M33.2	PM	145/170	85.3 (79.1, 90.3)	25.4 (21.9, 29.2)	0	0.0	0.0
M60.9	Myositis, unspecified	195/293	66.6 (60.8, 71.9)	34.2 (30.3, 38.3)	9	+1.40	+0.3
All codes	All above codes	509/651	78.2 (74.8, 81.3)	89.3 (86.5, 91.7)	21	+2.3	0.0

The exclusion of children substantially increases the PPV of M60.8, slightly increases the PPV of M60.9 and has little or no impact on sensitivity or PPV of other codes.

* Sensitivity refers to the ability of the code to pick up all cases of idiopathic inflammatory myopathy within the whole cohort regardless of the idiopathic inflammatory myopathy phenotype.

N/A: not applicable; PPV: positive predictive value.

Owing to the relative rarity of IIM and diagnostic codes for IIM within HES, the specificity and negative predictive value were 100.0% for all codes and combinations tested. The PPV, sensitivity and 95% CIs of each individual primary code are presented in Table 1. M36.0, coding for paraneoplastic PM/DM, was the highest PPV, at 100.0%. M60.9, coding for myositis unspecified, had the lowest PPV of 66.6%.

### Code combinations

The sensitivity and specificity of each code combination are given in Table 2. Looking at DM codes only (combination 4) was highly accurate, with PPV 94.8%, albeit insensitive (38.6%). High-performing combinations included combination 8, which gave a PPV of 92.1% whist retaining a sensitivity of 77.9%, or code 10, which gave a PPV of 91.1% with a marginally better sensitivity of 78.9%.

**Table 2.** Description of attempted code combinations and their performance for positive predictive value and sensitivity

Code combination number	Code combination description	Frequency	PPV (%) (95% CI)	Sensitivity (%) (95% CI)
1	Any code	651	78.2 (74.8–81.3)	89.3 (86.5–91.7)
2	Any code excluding least specific code M60.9	522	89.3 (86.3–91.8)	81.8 (78.3–84.8)
3	Two or more different IIM codes occurring together	299	94.3 (91.1–96.7)	49.5 (45.3–53.7)
4	DM codes only (M33.1 or M33.9)	232	94.8 (91.1–97.3)	38.6 (34.6–42.7)
5	ILD code in addition to any IIM code	137	92.70 (87.0–96.4)	22.30 (18.9–25.9)
6	Any IIM code with PPV > 90 (M36.0, M33.1, M33.9, M33.0 and G72.4)	402	93.3 (90.4–95.5)	65.8 (61.7–69.7)
7	Any IIM code with PPV > 90 or two or more IIM codes occurring together	450	92.9 (90.2–95.1)	73.3 (69.5–76.9)
8	Any IIM code with PPV > 90 or if IIM codes occur together or alongside an ILD code	482	92.1 (89.3–94.4)	77.9 (74.3–81.2)
9*	Any code except M60.9 unless it occurs alongside an ILD code	540	88.9 (85.9–91.4)	84.2 (81.0–87.1)
10	Any code except M60.9 or M33.2 unless they occur together or alongside an ILD code	494	91.1 (88.2–93.5)	78.9 (75.4–82.2)

Results include patients identified through national extension of the hospital episode statistics (HES) search and exclude children <16 years of age except M33.0 code.

* Code 9 is our optimal recommended algorithm, balancing PPV, sensitivity and clinical judgement.

ILD: interstitial lung disease; IMM: idiopathic inflammatory myopathy; PPV: positive predictive value.

### Exclusion codes

Of 27 proposed exclusion codes, 15 codes (121 patients) were observed to occur concomitantly with diagnostic codes for IIM (Table 2). Nevertheless, 84 of 121 (69.4%) of these were still confirmed to have IIM. Two codes (G71.1, coding for myotonic disordersm, and G73.7, coding for myopathy in other diseases classified elsewhere) identified only false positives but related to only one patient (Supplementary Table S2, available at *Rheumatology Advances in Practice* online). Four codes identified only true positives. Four of four patients with G72.0 (drug-induced myopathy) all had a statin-induced myositis, fitting within the autoimmune IIM spectrum. Three other codes with 100% PPV had only one patient per code.

### Interstitial lung disease

Including J99.1, J84.9 and J84.1 codes for co-existent lung disease improved PPV to 92.7% (combination 5). Sensitivity dropped to only 22.3%, accounting for the fact that not all IIM patients suffer from this complication. Of the 571 patients confirmed to have myositis, 143 (25.0%) also had IIM-associated ILD confirmed in their clinical records. Co-occurring ILD codes identified patients with IIM-associated ILD from the whole KCH population with a sensitivity of 70.6% and PPV of 78.8% (Table 3). When considering only the patients identified within HES by code 9, sensitivity within this cohort was 83.7%.

**Table 3.** Positive predictive value and sensitivity of ICD-10 codes associated with interstitial lung disease for detecting patients with idiopathic inflammatory myopathy-associated interstitial lung disease

ICD-10 code	Description	Frequency (*n*)	PPV to detect ILD (%) (95% CI)	Sensitivity (%) (95% CI)	Sensitivity within algorithm 9 (%)
J84.1	Other interstitial pulmonary diseases with fibrosis	88	83.0	47.7	56.6
J84.9	Interstitial pulmonary disease, unspecified	89	86.5	50.3	59.7
J99.1	Respiratory disorders in other diffuse CTD	66	80.3	34.6	41.1
All ILD codes	All above codes	137	78.8 (71.0–85.3)	70.6 (62.7–77.7)	83.7

ILD: interstitial lung disease; IMM: idiopathic inflammatory myopathy; PPV: positive predictive value.

### Sensitivity analyses

Five patients had insufficient notes available to confirm or refute a diagnosis. Sensitivity analysis assuming that all had IIM increased the PPV by 0.8% and sensitivity by 0.1%. Total admissions will include multiple admissions for some patients, thereby over-estimating the local population; therefore, calculations were repeated using the estimated local population served by the Trust according to the Care Quality Commission report: 1 000 000 people [18]. All specificities remained unchanged at 100.0%.

### Pattern of errors

Twenty-five of 142 false positives were being investigated appropriately for IIM at the time of coding, but ultimately considered by the clinical team not to have IIM. Other recurring or avoidable coding misdiagnoses identified included infective myositides (*n* = 20) and sarcoid myositis, rhabdomyolysis, metabolic myopathies, drug-induced myopathy, sickle cell crises, muscular dystrophies and PMR (*n* < 10 each).

Sixty-one locally known patients were not picked up using any IIM code on HES (false negatives). Mortality was higher in the true-positive group compared with the false-negative group [mortality rate ratio 3.6 (95% CI 1.4, 13.7), *P* < 0.05], implying that false negatives either suffer milder disease or are at an earlier time point in their illness. The mean age was lower in the false negatives (54.3 *vs* 61.4 years, *P* < 0.05). The percentage of males was the same (34 *vs* 32%, *P* = 0.66).

### Revalidation in another NHS Trust

The results were validated against a randomly generated sample of 60 HES records with IIM ICD-10 codes at Nottingham University Hospitals NHS Trust, a teaching hospital with specialized rheumatology services but without a specific myositis service (Supplementary Table S3, available at *Rheumatology Advances in Practice* online). In this cohort, only 36 of 60 (60.0%) records with diagnostic codes for IIM were confirmed at case note review to have IIM. Four children were then excluded, none of whom had true IIM. Combination 9 performed best, with a PPV of 94.4% (95% CI 81.3, 99.3), although all combinations had a PPV > 90% and overlapping confidence intervals. Within the study population of 60 patients, combination 9 picked up 34 of 36 true positives. Nine of these patients also received an ILD code, with a PPV of 77.8% for detecting IIM-associated ILD.

## Discussion

### Individual codes

Table 1 demonstrates how PPV of the individual ICD-10 codes varies from 100.0% (M36.0, coding for paraneoplastic PM/DM) to 66.6% (M60.9, coding for myositis, unspecified). Surprisingly, M33.2, the ICD-10 code for PM, was one of the worst-performing codes, with a PPV of only 85.3%. The lower PPV of M33.2 might be explained by the non-specific nature of PM symptoms and features, meaning that people with proximal weakness and/or high creatine kinase might initially be labelled as PM pending further investigation, whereas in fact there is a broad differential, and diagnosis might later be revised. This is in contrast to ICD-10 code M33.9 for DM, where some of the associated rashes are pathognomonic, meaning that M33.9 had a correspondingly had a high PPV of 95.5%.

### Optimal code combination

Combination PPVs of ∼90% mean that ICD-10 codes in HES could be a valuable method of identifying patients with IIM for further nationwide epidemiological research. The relative importance of sensitivity *vs* specificity of a test varies according to the specific research question, but algorithms identified within this study can be used according to researcher preference. To maximize case ascertainment, all listed ICD-10 codes for IIM should be used, but to optimize the search strategy and reduce false positives while minimizing the impact on case ascertainment, we would suggest excluding codes given while <16 years old (except for JDM code M33.0) and including all diagnostic codes for IIM except for M60.9, unless M609 occurs alongside an ILD code (combination 9; Table 2). This provides a PPV of 88.9% with a sensitivity of 84.2%. Although combination 10 has a higher PPV, it is counter-intuitive to exclude PM code M33.2 from future research about IIM.

The numbers involved in our validation are too small to recommend the use of any exclusion codes. On a national level, these extremely rare codes/conditions are unlikely to affect sufficient people to affect the PPV or sensitivity substantially. Where accuracy of diagnosis is important to the study, researchers could consider looking at DM codes only, which together had a PPV of 94.8%, although this will pick up only an estimated 39% of IIM patients and would bias towards a particular subset (combination 2). Alternatively, looking at those where two or more codes have been seen in a single patient also gives a PPV of 94.3% with a sensitivity of 49.5% (combination 3).

There are too many negative controls to make NPV and specificity data interpretable. Owing to the rarity of IIMs, the low incidence of admissions with IIM codes compared with the total number of admissions in the Trust creates an extremely low pre-test probability, making the specificity 100.0% for every code and combination tested. If codes were applied to a different population, for example attendees at a rheumatology clinic, the specificity and NPV would reduce.

RECORDER have separately looked at random cases across two other Trusts and found that 36 of 40 with codes M331 or M339 (PPV 90%), 22 of 38 with codes M332, M609, G724 or M608 (PPV 57.9%) and 15 of 15 with code M330 were genuine cases of IIM, reflecting our own findings of M609, M608 and M332 as the least accurate codes [19].

A larger proportion of HES-identified patients were dropped by combination 9 at the non-specialist centre (33 *vs* 24%). This reflects that proportionally fewer cases of this rare disease are likely to be referred to non-specialist centres; therefore, similar presenting features are more likely to signify an alternative underlying muscular condition (i.e. pre-test probability is lower). At the specialist centre, pre-test probability is higher, meaning that PPV is higher. Once low-probability patients are filtered out by combination 9, PPV is more comparable between the two centres (94.4 *vs* 89.9%).

### Interstitial lung disease

ILD is a common manifestation of IIMs. Twenty-five per cent of the KCH cohort were confirmed to have IIM-associated ILD, in keeping with estimates from other studies [20]. The J99.1, J84.1 and J84.9 codes were able to identify a reasonable proportion of these (71%). The sensitivity from within the population created by combination 9 is >80%, which would be hard to improve upon. ILD is an underdiagnosed complication, and mild forms might remain undetected or poorly recorded in clinical notes; therefore, the true prevalence within our retrospective study population is likely to be higher [21].

### Missing patients

Given the complexity of diagnosis and treatment of IIM, all patients with true IIM should have been seen in secondary care at some point in their illness. Many will have had at least one inpatient or day-case admission (acute illness during diagnosis or flares, muscle biopsy, infusion therapies, or from related/unrelated co-morbidities), creating the potential to be identified via HES hospital admission data.

Using combination 9, 16% of IIM patients in the community of a specialist centre were missed. Comparatively, including all codes (code 1) misses only 11% of cases but gives a false-discovery rate of 22%. Any prevalence estimates extrapolated from national HES data must be interpreted in light of false-discovery and false-negative rates. These estimates have not been validated in other populations.

Future researchers using the methods described also need to be conscious of the unsurprising bias towards more complex cases.

### Strengths and limitations

It has previously been shown that coded inpatient HES data can be a reliable and accurate data resource in certain conditions [22, 23]. This study adds that they are also valuable for IIM if specified coding algorithms are applied. Accuracy of HES coding has improved with time [23]. The introduction of payment by results in 2002, meaning that financial reimbursement of NHS Trusts was linked to coded performance data, created a drive to improve coding standards across the UK. Payment by results improved primary diagnosis coding accuracy from 73.8 to 96% [23]. To ensure modern data standards, only patients receiving codes since 2010 were included in the present study.

Information governance, including accuracy of HES data, is a key element examined regularly by the Care Quality Commission, meaning that HES coding is quality assured across England.

Our data reflect patients at a tertiary NHS Trust where local databases of IIM patients are maintained. The local database offered a unique advantage that patients within the hospital catchment population have been identified independently, allowing estimation of sensitivity and analysis of missing patients. Testing within a second Trust with a different population suggests that there is no major variation in coding practices between Trusts. Validation of sensitivity analyses could not be repeated within the second Trust owing to lack of data on the local background IIM population. Other groups from Canada and Sweden have previously used administrative data codes in IIM research, citing PPVs of >85%; however, methodologies are scantily described, sensitivity is not addressed, and older and more limited ICD-9 codes are used [22, 24, 25]. Although we have provided an estimate of sensitivity, the local database will not be complete. All quoted sensitivities are therefore likely to be over-estimates. However, to our knowledge, our local database is a unique resource, with no more-suitable alternative available. Efforts were made previously to increase the accuracy of the local database through screening all positive Myositis Specific Antibody results at the Trust for additional cases unknown to rheumatology/dermatology outpatients where the database was created. Our work demonstrates that single ICD-10 IIM codes in HES are frequently inaccurate; however, with application of the suggested algorithms, accuracy can be improved to increase the validity of future research.

In clinical studies, Peter and Bohan criteria or 2017 EULAR classification criteria are often used to confirm an IIM diagnosis and define the phenotypic subgroup [26]. In practice, diagnosis is a grey area, with wide phenotypic variation and many patients not falling into official PM, DM or IBM classifications; therefore, categorization of IIM patients remains topical [13]. The present study highlights that current ICD-10 coding categories do not clearly capture different subgroups of IIM. IBM, anti-synthetase syndrome, CTD-overlap syndromes, clinically amyopathic DM, immune-mediated necrotizing myopathies, JDM, PM and DM can all present and behave differently, and improving categorization would aid further research. Given the limited information provided by current ICD-10 definitions and the sparsity of detail in retrospective note review, there is insufficient information to confirm concordance with classification criteria or to divide patients accurately into subgroups. Pragmatically, our data therefore reflect clinical diagnoses of IIM, perhaps more accurately reflecting day-to-day patient and clinician experience.

### Conclusions

Administrative NHS data are potentially powerful epidemiological resources for IIM research that are already in use despite previously limited validation work in this field. Completeness of data in clinical registries is dependent on engagement of clinicians and patients to support data submission, whereas HES provides total coverage of a national population using existing data collection methods. The high PPV and sensitivity of the coding algorithms identified by this study make them more suitable for use in further epidemiological studies in IIMs than single ICD-10 codes. Taking a multi-source approach to registration of patients with IIM would result in a more complete cohort, but the methods described could be a key step towards creating a national registry for IIM. Identification of these patients is not only helpful for recruitment to clinical research but would also be useful to NHS England when planning specialized service provision for IIM. Methods could also be adapted to other under-resourced, rare diseases to assist cost-effective epidemiological research.

## Supplementary Material

rkac102_Supplementary_Data

## Data Availability

The data underlying this article cannot be shared publicly due to the confidential nature of health records. Data are available only to those who have the legal basis to access it, either through the Data Access Request Service (https://digital.nhs.uk/services/data-access-request-service-dars) or partnership with National Disease Registration Service.

## References

[1] Directorate-General for Health and Food Safety. Communication from the Commission COMM (2008) 679 final to the European Parliament, The Council, The European Economic and Social Committee and the Committee of the Regions on Rarer Diseases: Europe's challenges. 2008. https://eur-lex.europa.eu/legal-content/EN/TXT/?uri=celex%3A52008DC0679 (8 December 2022, date last accessed).

[2] Nguengang Wakap S , Lambert DM, Olry A et al Estimating cumulative point prevalence of rare diseases: analysis of the Orphanet database. Eur J Hum Genet 2020;28:165–73. 31527858 10.1038/s41431-019-0508-0 PMC6974615

[3] Department of Health and Social Care. The UK rare diseases framework. 2021:3–4. https://www.gov.uk/government/publications/uk-rare-diseases-framework.

[4] Dalakas MC. Polymyositis, dermatomyositis, and inclusion-body myositis. New Engl J Med 1991;325:1487–98. 10.1056/NEJM199111213252107 1658649

[5] Buchbinder R , Forbes A, Hall S, Dennett X, Giles G. Incidence of malignant disease in biopsy-proven inflammatory myopathy. A population-based cohort study. Ann Intern Med 2001;134:1087–95. 11412048 10.7326/0003-4819-134-12-200106190-00008

[6] Kiely PD , Chua F. Interstitial lung disease in inflammatory myopathies: clinical phenotypes and prognosis. Curr Rheumatol Rep 2013;15:359. 23888366 10.1007/s11926-013-0359-6

[7] Meyer A , Meyer N, Schaeffer M et al Incidence and prevalence of inflammatory myopathies: a systematic review. Rheumatology (Oxford) 2015;54:50–63. 25065005 10.1093/rheumatology/keu289

[8] Hospital Episode Statistics. About the HES database. https://digitalnhsuk/data-and-information/data-tools-and-services/data-services/hospital-episode-statistics (6 June 2022, date last accessed).

[9] Williams J , Mann R. Hospital episode statistics: time for clinicians to get involved? Clin Med 2002;2:34–7. 10.7861/clinmedicine.2-1-34 PMC4953168 11871636

[10] NHS Digital. National clinical coding standards ICD-10, 5th Edition (2021). 2021. https://classbrowser.nhs.uk/ref_books/ICD-10_2021_5th_Ed_NCCS.pdf (8 December 2022, date last accessed).

[11] NHS Digital. National clinical coding training handbook. The Health and Social Care Information Centre; 2017–2018. https://hscic.kahootz.com/gf2.ti/f/762498/27838725.1/PDF/-/NationalClinicalCodingTrainingHandbook1718.pdf (8 December 2022, date last accessed).

[12] Pearce F , Rutter M, Griffiths B et al O36 Validation of methods to enable national registration for rare autoimmune rheumatic diseases. Rheumatology (Oxford) 2020;59:59.

[13] Parker MJS , Oldroyd A, Roberts ME et al Increasing incidence of adult idiopathic inflammatory myopathies in the City of Salford, UK: a 10-year epidemiological study. Rheumatol Adv Pract 2018;2:rky035. 31431976 10.1093/rap/rky035 PMC6649983

[14] Dobloug C , Garen T, Bitter H et al Prevalence and clinical characteristics of adult polymyositis and dermatomyositis; data from a large and unselected Norwegian cohort. Ann Rheum Dis 2015;74:1551–6. 24695011 10.1136/annrheumdis-2013-205127

[15] Bossuyt PM , Reitsma JB, Bruns DE et al; STARD Group. STARD 2015: an updated list of essential items for reporting diagnostic accuracy studies. BMJ 2015;351:h5527. 26511519 10.1136/bmj.h5527 PMC4623764

[16] RStudio Team. RStudio: integrated development for R. Boston, MA: RStudio, PBC, 2020.

[17] StataCorp. Stata statistical software: release 17. College Station, TX: StataCorp, 2021.

[18] CQ Commission. King's College Hospital Quality Report. 13 October 2016.

[19] Peach E , Rutter M, Lanyon P et al Risk of death among people with rare autoimmune diseases compared with the general population in England during the 2020 COVID-19 pandemic. Rheumatology (Oxford) 2021;60:1902–9. 33271595 10.1093/rheumatology/keaa855 PMC7798585

[20] Lega J-C , Reynaud Q, Belot A et al Idiopathic inflammatory myopathies and the lung. Eur Respir Rev 2015;24:216–38. 26028634 10.1183/16000617.00002015 PMC9487811

[21] Hannah J , Gunawardena H. Picking interstitial lung disease out of the myositis haystack. Ind J Rheumatol 2020;15:91–8.

[22] Bernatsky S , Linehan T, Hanly JG. The accuracy of administrative data diagnoses of systemic autoimmune rheumatic diseases. J Rheumatol 2011;38:1612–6. 21532057 10.3899/jrheum.101149

[23] Burns EM , Rigby E, Mamidanna R, Bottle A et al Systematic review of discharge coding accuracy. J Public Health (Oxf) 2012;34:138–48. 21795302 10.1093/pubmed/fdr054 PMC3285117

[24] Dobloug GC , Svensson J, Lundberg IE, Holmqvist M. Mortality in idiopathic inflammatory myopathy: results from a Swedish nationwide population-based cohort study. Ann Rheum Dis 2018;77:40–7. 28814428 10.1136/annrheumdis-2017-211402

[25] Helmers SB , Jiang X, Pettersson D et al Inflammatory lung disease a potential risk factor for onset of idiopathic inflammatory myopathies: results from a pilot study. RMD Open 2016;2:e000342. 28123774 10.1136/rmdopen-2016-000342 PMC5237746

[26] Leclair V , Lundberg IE. New myositis classification criteria—what we have learned since Bohan and Peter. Curr Rheumatol Rep 2018;20:18. 29550929 10.1007/s11926-018-0726-4 PMC5857275

