# Supplementary material for: Validation of methods to identify people with idiopathic inflammatory myopathies using hospital episode statistics
Source: Rheumatol Adv Pract. 2022 Dec 2;6(3):rkac102. doi: 10.1093/rap/rkac102 (PMC9749128; doi:10.1093/rap/rkac102)
Supplement: rkac102_Supplementary_Data [file rkac102_supplementary_data.docx]

**Supplementary Data**

*Supplementary Table S1. The ICD-10 codes and their brief descriptions that were agreed by expert group consensus to be collected for analysis.*

| Codes to be considered as diagnostic codes for IIM | |
| --- | --- |
| M36.0 | Paraneoplastic PM/DM |
| M33.1 | Other dermatomyositis |
| M33.9 | Dermatomyositis |
| M33.0 | JDM |
| G72.4 | Inflammatory myopathy |
| M33.2 | Polymyositis |
| M60.8 | Other myositis |
| M60.9 | Myositis, unspecified |
| ILD Codes to be considered for Inclusion | |
| J84.1 | Other interstitial pulmonary diseases with fibrosis |
| J84.9 | Interstitial pulmonary disease, unspecified |
| J99.1 | Respiratory disorders in other diffuse connective tissue disorder |
| Codes to be considered for Exclusion | |
| D86.8 | Sarcoid Myositis |
| M60.0 | Interstitial Myositis |
| M63.3 | Myositis in Sarcoidosis |
| M63.8 | Other Disorders of Muscle In diseases classified elsewhere |
| G71.0 | Muscular dystrophy |
| G71.1 | Myotonic disorders |
| G71.3 | Mitochondrial myopathy, not elsewhere classified |
| G71.8 | Other primary disorders of muscles – includes rhabdomyolysis |
| G72.0 | Drug induced myopathy |
| G72.2 | Myopathy due to other toxic agents |
| G73.4 | Myopathy in infectious and parasitic disease classified elsewhere |
| G73.6 | Myopathy in metabolic disease |
| G73.7 | Myopathy in other diseases classified elsewhere |
| G72.8 | Other specified myopathies |
| G72.9 | Myopathy, unspecified |

*Supplementary Figure S1 – 2x2 tables*

**Individual Codes - KCH**

| J991 | Positive | Negative | Total |
| --- | --- | --- | --- |
| IIM | 63 | 507 | 570 |
| Non-IIM | 3 | 139 | 142 |
| Total | 66 | 646 | 712 |

| J841 | Positive | Negative | Total |
| --- | --- | --- | --- |
| IIM | 78 | 492 | 570 |
| Non-IIM | 10 | 132 | 142 |
| Total | 88 | 624 | 712 |

| J849 | Positive | Negative | Total |
| --- | --- | --- | --- |
| IIM | 85 | 485 | 570 |
| Non-IIM | 4 | 138 | 142 |
| Total | 89 | 623 | 712 |

| G724 | Positive | Negative | Total |
| --- | --- | --- | --- |
| IIM | 193 | 377 | 570 |
| Non-IIM | 17 | 125 | 142 |
| Total | 210 | 502 | 712 |

| M330 | Positive | Negative | Total |
| --- | --- | --- | --- |
| IIM | 19 | 551 | 570 |
| Non-IIM | 1 | 141 | 142 |
| Total | 20 | 692 | 712 |

| M331 | Positive | Negative | Total |
| --- | --- | --- | --- |
| IIM | 202 | 368 | 570 |
| Non-IIM | 10 | 132 | 142 |
| Total | 212 | 500 | 712 |

| M332 | Positive | Negative | Total |
| --- | --- | --- | --- |
| IIM | 145 | 425 | 570 |
| Non-IIM | 25 | 117 | 142 |
| Total | 170 | 542 | 712 |

| M339 | Positive | Negative | Total |
| --- | --- | --- | --- |
| IIM | 84 | 486 | 570 |
| Non-IIM | 4 | 138 | 142 |
| Total | 88 | 624 | 712 |

| M608 | Positive | Negative | Total |
| --- | --- | --- | --- |
| IIM | 82 | 488 | 570 |
| Non-IIM | 10 | 132 | 142 |
| Total | 92 | 620 | 712 |

| M609 | Positive | Negative | Total |
| --- | --- | --- | --- |
| IIM | 195 | 375 | 570 |
| Non-IIM | 98 | 44 | 142 |
| Total | 293 | 419 | 712 |

| M360 | Positive | Negative | Total |
| --- | --- | --- | --- |
| IIM | 10 | 560 | 570 |
| Non-IIM | 0 | 142 | 142 |
| Total | 10 | 702 | 712 |

| Any ILD code | Positive | Negative | Total |
| --- | --- | --- | --- |
| IIM | 127 | 443 | 570 |
| Non-IIM | 10 | 132 | 142 |
| Total | 137 | 575 | 712 |

**Code Combinations - KCH**

| Code 1 | Positive | Negative | Total |
| --- | --- | --- | --- |
| IIM | 509 | 61 | 570 |
| Non-IIM | 142 | 0 | 142 |
| Total | 651 | 61 | 712 |

| Code 2 | Positive | Negative | Total |
| --- | --- | --- | --- |
| IIM | 466 | 104 | 570 |
| Non-IIM | 56 | 86 | 142 |
| Total | 522 | 190 | 712 |

| Code 3 | Positive | Negative | Total |
| --- | --- | --- | --- |
| IIM | 282 | 288 | 570 |
| Non-IIM | 17 | 125 | 142 |
| Total | 299 | 413 | 712 |

| Code 4 | Positive | Negative | Total |
| --- | --- | --- | --- |
| IIM | 220 | 350 | 570 |
| Non-IIM | 12 | 130 | 142 |
| Total | 232 | 480 | 712 |

| Code 5 | Positive | Negative | Total |
| --- | --- | --- | --- |
| IIM | 127 | 443 | 570 |
| Non-IIM | 10 | 132 | 142 |
| Total | 137 | 575 | 712 |

| Code 6 | Positive | Negative | Total |
| --- | --- | --- | --- |
| IIM | 375 | 195 | 570 |
| Non-IIM | 27 | 115 | 142 |
| Total | 402 | 310 | 712 |

| Code 7 | Positive | Negative | Total |
| --- | --- | --- | --- |
| IIM | 418 | 152 | 570 |
| Non-IIM | 32 | 110 | 142 |
| Total | 450 | 262 | 712 |

| Code 8 | Positive | Negative | Total |
| --- | --- | --- | --- |
| IIM | 444 | 126 | 570 |
| Non-IIM | 38 | 104 | 142 |
| Total | 482 | 230 | 712 |

| Code 9 | Positive | Negative | Total |
| --- | --- | --- | --- |
| IIM | 480 | 90 | 570 |
| Non-IIM | 60 | 82 | 142 |
| Total | 540 | 172 | 712 |

| Code 10 | Positive | Negative | Total |
| --- | --- | --- | --- |
| IIM | 450 | 120 | 570 |
| Non-IIM | 44 | 98 | 142 |
| Total | 494 | 218 | 712 |

**ILD at KCH results – ability to detect IIM-ILD**

| J991 | Positive | Negative | Total |
| --- | --- | --- | --- |
| IIM-ILD | 53 | 100 | 153 |
| No ILD | 13 | 546 | 559 |
| Total | 66 | 646 | 712 |

| J841 | Positive | Negative | Total |
| --- | --- | --- | --- |
| IIM-ILD | 73 | 80 | 153 |
| No ILD | 15 | 544 | 559 |
| Total | 88 | 624 | 712 |

| J849 | Positive | Negative | Total |
| --- | --- | --- | --- |
| IIM-ILD | 77 | 76 | 153 |
| No ILD | 12 | 547 | 559 |
| Total | 89 | 623 | 712 |

| Any ILD Code | Positive | Negative | Total |
| --- | --- | --- | --- |
| IIM-ILD | 108 | 45 | 153 |
| No ILD | 29 | 530 | 559 |
| Total | 137 | 575 | 712 |

**Nottingham Revalidation**

| Code 1 | Positive | Negative | Total |
| --- | --- | --- | --- |
| IIM | 36 | N/A | 36 |
| Non-IIM | 20 | N/A | 20 |
| Total | 56 | N/A | 56 |

| Code 2 | Positive | Negative | Total |
| --- | --- | --- | --- |
| IIM | 33 | 3 | 36 |
| Non-IIM | 2 | 18 | 20 |
| Total | 35 | 21 | 56 |

| Code 3 | Positive | Negative | Total |
| --- | --- | --- | --- |
| IIM | 11 | 25 | 36 |
| Non-IIM | 1 | 19 | 20 |
| Total | 12 | 44 | 56 |

| Code 4 | Positive | Negative | Total |
| --- | --- | --- | --- |
| IIM | 14 | 22 | 36 |
| Non-IIM | 1 | 19 | 20 |
| Total | 15 | 41 | 56 |

| Code 5 | Positive | Negative | Total |
| --- | --- | --- | --- |
| IIM | 9 | 27 | 36 |
| Non-IIM | 0 | 20 | 20 |
| Total | 9 | 47 | 56 |

| Code 6 | Positive | Negative | Total |
| --- | --- | --- | --- |
| IIM | 27 | 9 | 36 |
| Non-IIM | 1 | 19 | 20 |
| Total | 28 | 28 | 56 |

| Code 7 | Positive | Negative | Total |
| --- | --- | --- | --- |
| IIM | 29 | 7 | 36 |
| Non-IIM | 2 | 18 | 20 |
| Total | 31 | 25 | 56 |

| Code 8 | Positive | Negative | Total |
| --- | --- | --- | --- |
| IIM | 31 | 5 | 36 |
| Non-IIM | 2 | 19 | 20 |
| Total | 33 | 23 | 56 |

| Code 9 | Positive | Negative | Total |
| --- | --- | --- | --- |
| IIM | 34 | 2 | 36 |
| Non-IIM | 2 | 18 | 20 |
| Total | 36 | 20 | 56 |

| Code 10 | Positive | Negative | Total |
| --- | --- | --- | --- |
| IIM | 33 | 3 | 36 |
| Non-IIM | 2 | 18 | 20 |
| Total | 35 | 21 | 56 |

Supplementary Table S2. Frequency of co-occurrence of exclusion codes and their association with true diagnosis of IIM

|  | Description | Frequency (n) | Cases with IIM | False Positive Rate | PPV |
| --- | --- | --- | --- | --- | --- |
| D86.8 | Sarcoid Myositis | 5 | 1 | 80% | 20.0% |
| M60.0 | Interstitial Myositis | 4 | 1 | 75% | 25.0% |
| M63.3 | Myositis in Sarcoidosis | 1 | 1 | 0% | 100.0% |
| M63.8 | Other Disorders of Muscle In diseases classified elsewhere | 57 | 35 | 39% | 61.4% |
| G71.0 | Muscular dystrophy | 5 | 3 | 40% | 60.0% |
| G71.1 | Myotonic disorders | 1 | 0 | 100% | 0.0% |
| G71.3 | Mitochondrial myopathy, not elsewhere classified | 6 | 5 | 17% | 83.3% |
| G71.8 | Other primary disorders of muscles – includes rhabdomyolysis | 2 | 1 | 50% | 50.0% |
| G72.0 | Drug induced myopathy | 4 | 4 | 0% | 100.0% |
| G72.2 | Myopathy due to other toxic agents | 1 | 1 | 0% | 100.0% |
| G73.4 | Myopathy in infectious and parasitic disease classified elsewhere | 1 | 1 | 0% | 100.0% |
| G73.6 | Myopathy in metabolic disease | 4 | 3 | 25% | 75.0% |
| G73.7 | Myopathy in other diseases classified elsewhere | 1 | 0 | 100% | 0.0% |
| G72.8 | Other specified myopathies | 30 | 27 | 10% | 90.0% |
| G72.9 | Myopathy, unspecified | 26 | 22 | 15% | 84.6% |
| Any exclusion code |  | 121 | 84 | 31% | 20.0% |

PPV: positive predictive value.

*Supplementary Table S3. Results of code combination algorithm in Nottingham cohort. n=56*

|  | PPV | True Positives | False negatives |
| --- | --- | --- | --- |
| Code 1 | N/A | 36 | N/A |
| Code 2 | 94.3  (80.3-99.3) | 33 | 3 |
| code 3 | 91.7  (61.5-99.8) | 11 | 25 |
| code 4 | 93.3  (68.1-99.8) | 14 | 22 |
| code 5 | 100  (66.4-100.0) | 9 | 27 |
| code 6 | 96.4  (81.7-99.9) | 27 | 9 |
| code 7 | 93.5  (78.6-99.2) | 29 | 7 |
| code 8 | 93.9  (79.8-99.3) | 31 | 5 |
| code 9 | 94.4  (81.3-99.3) | 34 | 2 |
| code 10 | 94.3  (80.8-99.3) | 33 | 3 |
